# Habitat modification and seasonality influence avian haemosporidian parasite distributions in southeastern Brazil

**DOI:** 10.1371/journal.pone.0178791

**Published:** 2017-06-02

**Authors:** Francisco C. Ferreira Junior, Raquel A. Rodrigues, Vincenzo A. Ellis, Lemuel O. Leite, Magno A. Z. Borges, Érika M. Braga

**Affiliations:** 1 Departamento de Parasitologia, Instituto de Ciências Biológicas, Universidade Federal de Minas Gerais, Belo Horizonte, MG, Brazil; 2 Departamento de Biologia Geral, Universidade Federal de Minas Gerais, Belo Horizonte, MG, Brazil; 3 Centro de Ciências Biológicas e da Saúde, Universidade Estadual de Montes Claros, Campus Universitário Professor Darcy Ribeiro, Montes Claros, MG, Brazil; Charles University, CZECH REPUBLIC

## Abstract

Habitat modification may change vertebrate and vector-borne disease distributions. However, natural forest regeneration through secondary succession may mitigate these effects. Here we tested the hypothesis that secondary succession influences the distribution of birds and their haemosporidian parasites (genera *Plasmodium* and *Haemoproteus*) in a seasonally dry tropical forest, a globally threatened ecosystem, in Brazil. Moreover, we assessed seasonal fluctuations in parasite prevalence and distribution. We sampled birds in four different successional stages at the peak and end of the rainy season, as well as in the middle and at the end of the dry season. A non-metric multidimensional scaling analysis revealed that bird communities in the pasture (i.e., highly modified) areas were different from those in the early, intermediate, and late successional areas (secondary forests). Among 461 individual birds, haemosporidian prevalence was higher in pasture areas than in the more advanced successional stages, but parasite communities were homogeneous across these areas. Parasite prevalence was higher in pasture-specialists birds (resilient species) than in forest-specialists species, suggesting that pasture-specialists may increase infection risk for co-occurring hosts. We found an increase in prevalence between the middle and end of the dry season, a period associated with the beginning of the breeding season (early spring) in southeastern Brazil. We also found effects of seasonality in the relative prevalence of specific parasite lineages. Our results show that natural forest recovery through secondary succession in SDTFs is associated with compositional differences in avian communities, and that advanced successional stages are associated with lower prevalence of avian haemosporidian parasites.

## Introduction

Land conversion for the purposes of livestock farming and agriculture development can alter vector-borne disease distributions [1,2]. Such changes can result in the loss of biodiversity [3,4] and an increase in the prevalence of infectious disease in humans [5,6] and wildlife [6,7]. As human populations grow, human-mediated environmental changes will continue to occur and will impact host-parasite interactions worldwide [8]. Therefore, it is of crucial importance to understand how habitat modifications affect the distributions of disease-causing organisms.

Natural forest regeneration may mitigate land conversion effects on vector-borne disease distributions. This process, known as secondary succession, results from the recolonization of a previously deforested area by short-lived pioneers plant species that are eventually substituted by long-lived species [9,10]. It often takes decades for secondary succession to reach the structure and complexity (e.g., tree height, density, and species number) of mature forests [11,12]. While there is some controversy regarding how efficient “secondary forests” are at sustaining long-term biodiversity [13], secondary succession undoubtedly changes faunal richness and composition [14] by increasing the local diversity of forest-dependent animals [15,16].

Since changes in vertebrate community structure can influence disease transmission [17–19], it is logical to suspect that pathogens distributions may change within areas undergoing secondary succession. For example, disturbed areas (i.e., those in the early stages of succession) can favor resilient species that are important pathogen reservoirs [15,20], thereby increasing pathogen transmission and disease risk [21,22]. Conversely, advanced successional stages, which harbor greater species diversity than early stages, may have lower pathogen prevalence [21,23].

Avian haemosporidians are vector-borne protozoan parasites, and two main genera are transmitted by different dipteran families: *Plasmodium* (Culicidae) and *Haemoproteus* (Hippoboscidae for the subgenus *Haemoproteus* and Ceratopogonidae for the subgenus *Parahaemoproteus*). These parasites are globally distributed, and can infect a wide number of host species [24,25]. The diversity of host species a parasite can infect is an important parasite phenotype known as “host breadth”. Avian haemosporidian host breadth may be labile over small geographic distances and temporal scales [26], potentially posing risks to naïve birds populations, especially in rapidly changing environments [27]. Furthermore, habitat modification in tropical forests has been associated with changes in prevalence and richness of these parasites in Cameroon [28,29], Ghana [30], Australia [31], and Costa Rica [32]. In Hawaii, for example, human activities such as agriculture and urbanization increase the risk of contact between vectors and susceptible avian hosts [33]. Such contacts are relevant because avian malaria have driven bird species and populations to extinction in Hawaii [34]. However, some birds populations can recover after local extinctions [35], showing that habitat quality can play a role on the recolonization of impacted areas by resistant individuals inhabiting adjacent forest fragments.

Seasonally dry tropical forest (SDTF) is one of the most threatened ecosystems in the world [36]. Most remaining areas of SDTF are found in the Americas, and more than 66% of these forests have been lost due to human activities [37]. The current distribution of SDTF along the American continent is mostly composed of mosaics of secondary forests at different successional stages that vary in habitat structure, biodiversity, and integrity of ecological functions [11,36]. One of the largest areas of SDTF is found in Brazil, but only small portions of its distribution are protected by Brazilian law [38]. The reestablishment of forests on abandoned pasturelands has become an ongoing process in some parts of Brazil [37], what has increased local plant diversity and structure [39,40], changing insect-plant interactions [41] and the distributions of vertebrates such as bats [42,43] and birds [42].

The objective of our work was to test whether secondary succession in SDTFs influences the distribution of avian haemosporidian parasites (*Plasmodium* and *Haemoproteus*). To do this, we measured the prevalence and diversity of avian haemosporidians in areas corresponding to different successional stages in a SDTF in southeastern Brazil. We predicted that the difference in forest structure between successional stages would change the composition of host communities, consequently changing parasite prevalence and composition among areas. Since STDFs have a marked deciduous phytophysiognomy, with up to 95% of leaf area lost during the dry season [39,40], we hypothesized that seasonality would affect haemosporidian distributions as well. Avian haemosporidians can exhibit seasonal changes in prevalence in temperate climates, with some parasite lineages having lower prevalence during the winter compared with spring and summer [44–46]. We therefore sampled parasites at the peak and end of the rainy season, as well as at the middle and end of the dry season.

## Materials and methods

### Study site

This study was conducted at the Mata Seca State Park (MSSP), a conservation site of 15,466 ha located in the São Francisco river valley in southeastern Brazil (14°48′36″ – 14°56′59″S and 43°55′12″ – 44°04′12″W). The region’s climate is classified as tropical with dry summers (“*As*” category in Köppen’s classification [47]), with an average temperature of 23.4°C. The dry season starts in May with an average rainfall of 6 mm and the four following months typically receive less than 10 mm of rain in total. The rainy season starts in October and peaks between December and January, with monthly precipitation around 200 mm [47]. The area has a history of extensive cattle grazing, and approximately 1,525 ha of the Mata Seca State Park consist of abandoned pasture fields at different successional stages, with the remaining areas being mosaics of primary and secondary patches of SDTF. These forests are dominated by deciduous trees that lose up to 95% of leaf area during the dry season [39,40].

**Fig 1:**
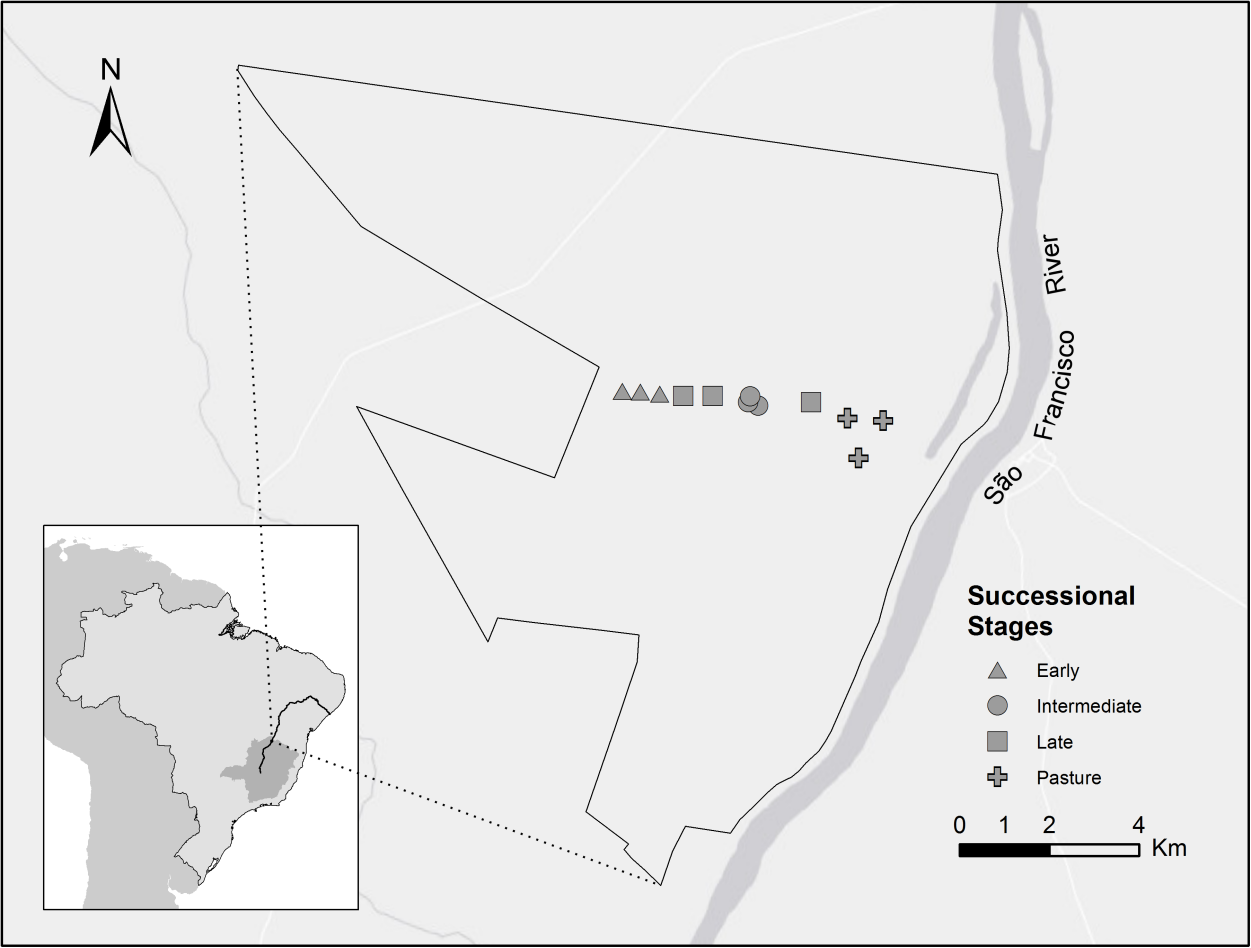
Map of Mata Seca State Park in Minas Gerais, Brazil, showing the sampling areas. Geographic coordinates from the sampling points: Early stages: 14°50'58.00"S, 44° 0'28.00"W; 14°50'57.00"S, 44°0'14.00"W; 14°50'57.11"S, 43°59'58.82"W. Intermediate stages: 14°50'58.00"S, 43°58'42.00"O; 14°50'56.21"S, 43°58'50.15"O; 14°50'52.00"S, 43°58'49.00" W. Late stages: 14°50'56.74"S, 43°59'40.51"W; 14°50'54.70"S, 14°50'51.79"S, 43°59'17.89"W; 43°58'1.53"W. Pasture areas: 14°51'27.09"S, 43°57'20.41"W; 14°51'0.35"S, 43°57'32.24"W; 14°50'59.42"S, 43°57'4.39"W.

We captured birds in four different successional stages inside the Mata Seca State Park, defined as “pasture”, “early stage”, “intermediate stage”, and “late stage” (Fig 1). We established three plots within each successional stage. The distance between plots within a successional stage varied from 0.13km to 2.27km, in an altitudinal range between 460 and 489 meters above sea level. The area defined here as “pasture” was used for extensive cattle grazing and was abandoned in 2008, five years before the beginning of our study. This vegetation consisted of exotic grass species, herbs and shrubs, and sparse trees. We defined pasture areas that were abandoned in the year 2000, 13 years before our study, as “early stage”. This successional stage had a single stratum of sparse trees, dominated by grasses, herbs, and shrubs. The “intermediate” stage represented pastures abandoned around 30 years before our study. This vegetation was composed of two strata of trees: deciduous trees of 10-12m in height and an understory of young trees, herbaceous species, and a high density of lianas. Finally, the “late stage” had no record of human intervention for at least the last 50 years. This successional stage also had two strata of trees: tall deciduous trees forming a closed canopy at 18-20m in height and a lower stratum of low-density young trees and lianas. Further details regarding phenological changes across secondary succession in the area can be found in Madeira et al. [36] and in Pezzini et al. [37].

We sampled birds at the end (April) of the rainy season in 2013, at the middle (June) and at the end (September) of the dry season in 2013, and at the peak of the rainy season (January) in 2014. Sampling effort was the same for all plots and sampling periods. We operated 15 mist-nets (12m long × 3m high, with 20mm mesh size; one set of mist-net per plot) for six hours starting at sunrise, and nets were checked every 30 minutes.

### Blood collection and DNA extraction

Captured birds were physically restrained and we obtained blood samples through brachial venipuncture. We stored the samples in absolute ethanol at room temperature for a maximum of five days and the material were ultimately kept at -20°C until DNA was extracted. Before release, birds were tagged with individual aluminum leg-rings provided by the Brazilian Research Center for the Conservation of Wild Birds (CEMAVE—license number 2965/4). This study was approved by the Ethics Committee in Animal Experimentation (CETEA), Universidade Federal de Minas Gerais, Brazil (Protocol #254/2011).

Approximately 10 μL of blood was transferred to 1.5 mL microtubes and samples were dried at 37°C for subsequent DNA extraction, for which we used a conventional phenol-chloroform method with isopropanol precipitation [48]. The genomic DNA pellet was resuspended in 50 μL of ultrapure water and quantified using a NanoDrop 2000 (Thermo Scientific, Waltham, United States).

### Molecular characterization of haemosporidians

Between 50 and 100 ng of the extracted DNA was used for a screening PCR that amplifies a 154-nucleotide segment of ribosomal RNA coding sequence within the mitochondrial DNA of *Plasmodium* and *Haemoproteus* in a single reaction. We used the primers 343F (5′-GCTCACGCATCGCTTCT-3′) and 496R (5′-GACCGGTCATTTTCTTTG-3′) designed by Fallon et al. [49] under PCR conditions and amplification analysis described by Roos et al. [50].

DNAs from positive individuals were submitted to a nested-PCR targeting the amplification of a 478 bp region of the cytochrome *b* gene. For the first reaction, we used primers HaemNFI (5′-AGACATGAAATATTATGGITAAG-3′) and HaemNR3 (5′-GAAATAAGATAAGAAATACCATTC-3′) [51] with 50–100 ng of genomic DNA. A 1-μL aliquot of this PCR product was then used as a template for the second reaction with the primers HaemF (5′-CTTATGGTGTCGA-TATATGCATG-3′) and HaemR2 (5′-CGCTTATCTGGAGATTGTAATGGTT-3′) [52]. DNA extracted from blood samples of chickens experimentally infected with *Plasmodium gallinaceum* and ultrapure water were used as positive and negative controls, respectively. These nested-PCRs followed the protocol in Hellgren et al. [51].

Products from all positive nested-PCRs were purified with Polyethylene Glycol 8000 [48] and bi-directionally sequenced with dye-terminator fluorescent labeling in an ABI Prism 3100 sequencer (Applied Biosystems, Foster City, United States). DNA sequences were aligned, checked for the presence of mixed infections (presence of double peaks in the eletrochromatograms) and edited using ChromasPro (Technelysium Pty Ltd, Helensvale, Australia), and compared with data available in the public databases Genbank and MalAvi [53]. We considered sequences as different cytochrome *b* lineages when they differed by one or more nucleotides. Lineages with no database records were considered novel and sequences were deposited in GenBank under accession numbers KP686094-KP686099, KP686101-KP686108 and KY346520. New records of previously described sequences were deposited in GenBank under accession numbers KY304994-KY305008.

A Bayesian phylogenetic tree for the parasite sequences found in this study was produced using MrBayes 3.2.2 [54] with the GTR + I + G model of nucleotide evolution, as recommended by ModelTest [55], which selects the best-fit nucleotide substitution model for a set of genetic sequences. We ran two Markov chains simultaneously for 5 million generations in total that were sampled every 1000 generations. The first 1250 trees (25%) were discarded as a burn-in step and the remaining trees were used to calculate the posterior probabilities of each estimated node in the final consensus tree. We used *Leucocytozoon schoutedeni* as the outgroup to root the phylogenetic tree.

### Statistical analysis

We used chi-square tests with Yates’ correction to assess the difference in prevalence between successional stages and sampling periods. For further analyses, we included only well-sampled host species, which we defined as species that were sampled seven or more times. We compared haemosporidian prevalence in habitat-specialist host species. Habitat-specialist host species were defined as species captured seven or more times exclusively in pasture (pasture-specialists) and species captured seven or more times exclusively in secondary forests (non-pasture areas), such as early, intermediate and late stage of succession (forest-specialists). For this comparison of prevalence, we pooled individual birds in the two habitat-specialist groups to calculate group-level haemosporidian prevalence. We used Fisher’s exact test when 2×2 frequency tables for prevalence had cell values smaller than five.

The relative abundances of bird species and parasite lineages across successional stages and seasons were used to construct Bray-Curtis dissimilarity matrices [56] and were ordinated by non-metric multidimensional scaling (NMDS). Differences between groups (i.e., successional stages and seasons) in multidimensional space were tested statistically by one-way analysis of similarity (ANOSIM). ANOSIM compares average Bray-Curtis dissimilarities within and between groups (e.g. successional stages). It produces an “R” statistic which is positive when average dissimilarities between groups are greater than average dissimilarities within groups. R approaches zero when average dissimilarities between and within groups are similar. R is tested for significance by permuting the grouping variable. The correlation between Bray-Curtis dissimilarities in birds and haemosporidians was assessed with a Mantel test using 9,999 permutations to test for significance. These multivariate analyses were conducted in the package vegan [57] in R v.3.3.1 [58].

## Results

### Bird distribution

**Fig 2:**
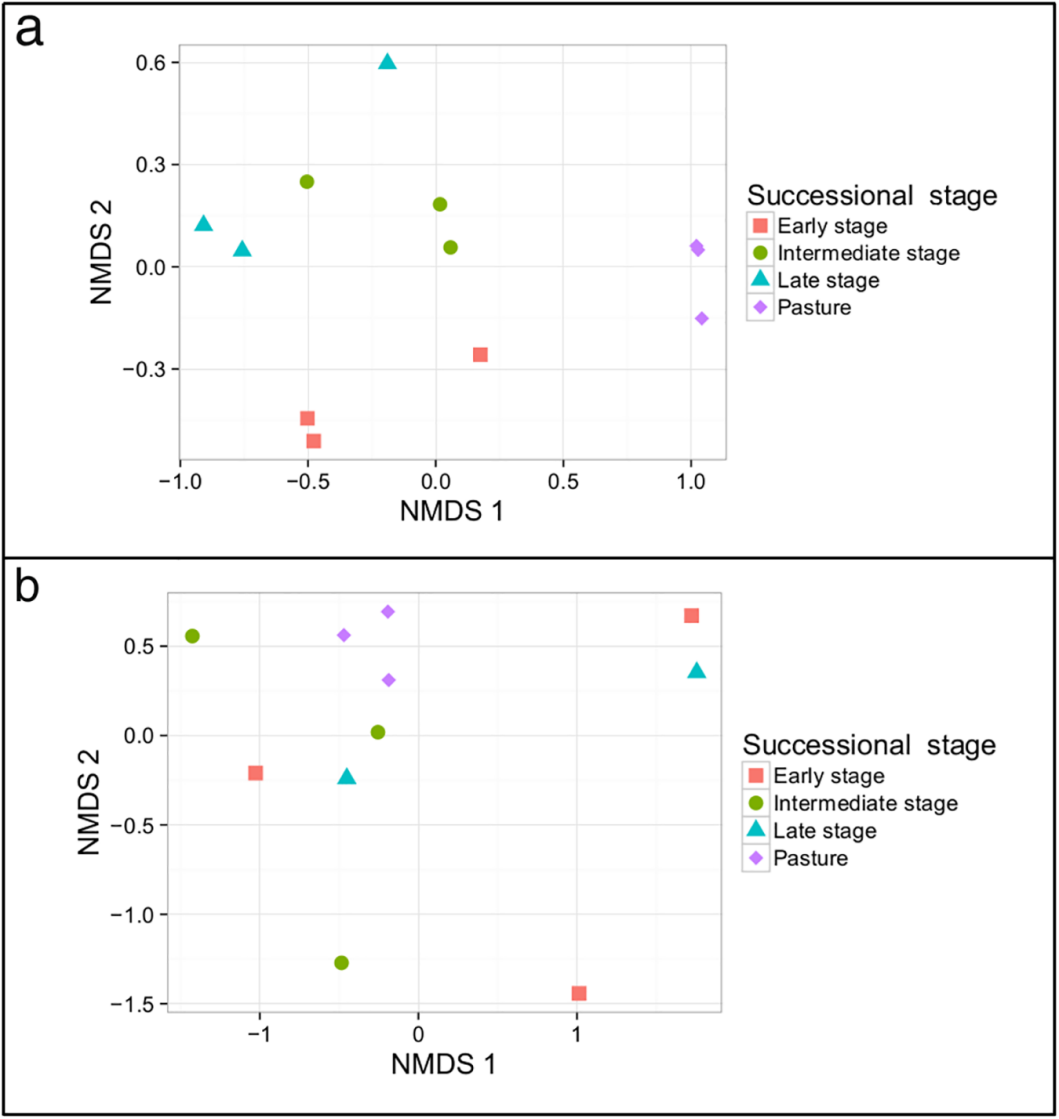
**Nonmetric multidimensional scaling (NMDS) showing bird (a) and parasite (b) community dissimilarities among successional stages.** Bird communities were dissimilar between pasture and non-pasture areas (ANOSIM; R = 0.98; P < 0.01), but there was no difference in parasite dissimilarity (ANOSIM; R = 0.1; P = 0.25).

We analyzed blood samples from 461 birds belonging to 5 orders, 21 families, and 64 species. Our NMDS analysis suggested that bird communities in the pasture areas were different than those in the other successional stages (ANOSIM; R = 0.98; *P* < 0.01; Fig 2A). In total, 20 bird species were sampled seven or more times, and we considered these as well-sampled species. Of those well-sampled species, two were restricted to pasture areas and eight were found only in the other three successional stages (secondary forests). The bird species *Volatinia jacarina* was captured 27 times in pasture areas and once in an early stage area. We therefore considered this species a pasture specialist in accordance with the species’ biology [59,60]. The single individual of *V*. *jacarina* found outside of the pasture area was removed from analyses comparing parasites of pasture-specialist and forest-specialist hosts only, but it was included in the community analysis for bird composition and parasite prevalence.

There was no difference in bird composition in relation to seasons (ANOSIM; R = -0.06; *P* = 0.68). From the 20 well-sampled species, *Coryphospingus pileatus* and *Thamnophilus pelzelni* were commonly found in all months, and *Myiopagis viridicata* (n = 19) was captured only in the rainy season. There were no species exclusively found in the dry season (S1 Table).

### General parasite prevalence and diversity

From 461 individuals screened, 193 (42%) from 46 species were positive for *Plasmodium* or *Haemoproteus* (Table 1). Prevalence among the 20 well-sampled host species ranged from 0% (*Conirostrum speciosum*; n = 7) to 75% (*Columbina squammata*; n = 12) (S2 Table). All 193 samples that screened positive were subjected to the cytochrome *b* PCR, which successfully amplified infections from 97 of the individuals that screened positive. However, high-quality sequences were obtained from only 81 of those 97 samples, with no mixed infections detected. *Plasmodium* parasites were detected in 63 birds, representing 19 unique lineages. *Haemoproteus* was detected in 18 birds, representing five unique lineages from the subgenus *Parahaemoproteus* and six unique lineages in the subgenus *Haemoproteus*. Overall, relative prevalence of each parasite group was: *Plasmodium* = 77.8%; *Parahaemoproteus =* 8.6% and *Haemoproteus =* 13.6%. A total of 15 lineages were detected here for the first time (seven *Plasmodium* and eight *Haemoproteus*, S3 Table).

**Table 1:** Number of birds sampled and haemosporidian prevalence by successional stage and season in the Mata Seca State Park.

	End rainy	Middle dry	End dry	Peak rainy	Total
Successional stage	n (%)	n (%)	n (%)	n (%)	n (%)
Pasture	34 (53%)	28 (46%)	44 (57%)	60 (58%)	**168 (55%)**
Early	27 (22%)	11 (27%)	29 (59%)	8 (37%)	**75 (39%)**
Intermediate	28 (29%)	39 (23%)	28 (46%)	23 (56%)	**118 (36%)**
Late	30 (33%)	24 (20%)	23 (26%)	23 (35%)	**100 (29%)**
Total	**119 (35%)**	**102 (29%)**	**126 (49%)**	**114 (52%)**	**461 (42%)**

n = number of sampled birds; % = prevalence of *Plasmodium/Haemoproteus* detected in the screening PCR.

### Parasites and habitat

**Fig 3:**
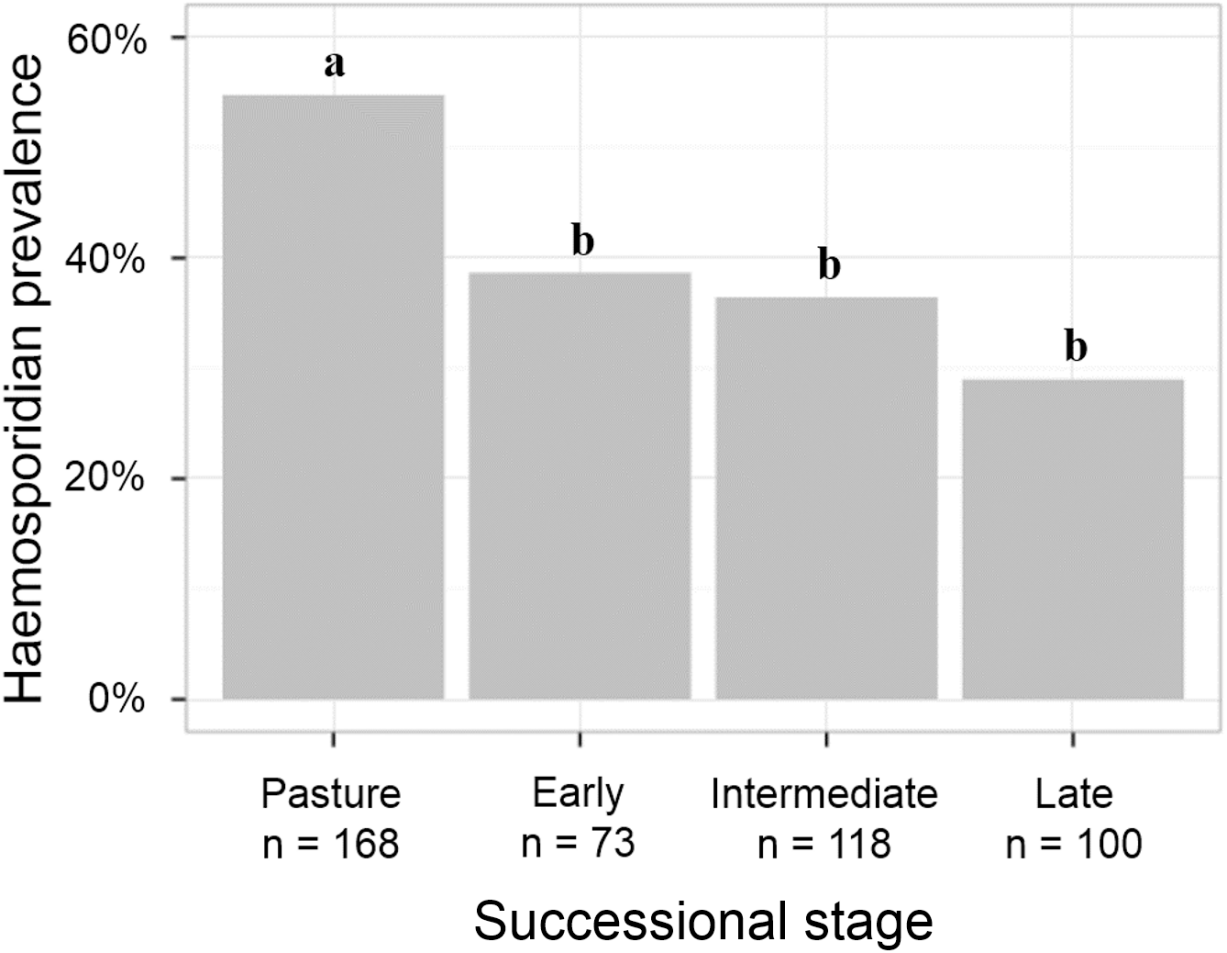
Haemosporidian prevalence across successional stages. Different letters above columns indicate statistically significant difference in prevalence between successional stages.

Overall prevalence differed across successional stages (χ^2^ test, *P* < 0.001, χ^2^ = 20, df = 3; Fig 3). In particular, prevalence was higher in the pasture than in any of the other three successional stages (pasture vs. early, χ^2^ test, *P* = 0.03, χ^2^ = 4.8, df = 1; pasture vs. intermediate, χ^2^ test, *P* < 0.01, χ^2^ = 8.62, df = 1; pasture vs. late, χ^2^ test, *P* = < 0.001, χ^2^ = 15.8, df = 1; Table 1); but there was no difference in prevalence between the non-pasture successional stages (χ^2^ test, *P* = 0.35, χ^2^ = 2.1, df = 2; Table 1). Prevalence was higher in pasture-specialists than in forest-specialists bird species (pasture = 58% (25/43); non-pasture = 35.3% (49/139); χ^2^ test, *P* = 0.01, χ^2^ = 6.2, df = 1, Table 2, S1 Fig). We analyzed *Coryphospingus pileatus* separately, since it was distributed across all successional stages, and there was no difference in prevalence among stages (58.8% (n = 90); χ^2^ test, *P* = 0.9, χ^2^ = 0.49, df = 3, S2 Table) or when comparing the pasture to non-pasture stages (χ^2^ test, *P* = 0.82, χ^2^ = 0.05, df = 1, Table 2).

**Table 2:** Haemosporidian prevalence and lineages distribution according to bird habitat use for habitat-specialists and generalist species.

	Bird species	Number of individuals (prevalence^a^)	Parasite lineages detected (Number of times detected^b^)
Pasture-specialists	*Ammodramus humeralis*	8 (62%)	*P*. *nucleophilum*, *P*. *cathemerium*
	*Columbina picui*	8 (62%)	h.h.COPIC01 (2)
	*Volatinia jacarina*	27 (55%)	p.BAFLA03 (3), p.PADOM11 (3), p.P-T138 (4)
Forest-specialists	*Basileuterus flaveolus*	8 (37%)	p.PHPAT01, p.BAFLA03
	*Cnemotriccus fuscatus*	10 (40%)	p.PADOM09, h.p.PAPOL02, p.BAFLA03
	*Formicivora melanogaster*	19 (42%)	p.FOMEL01 (2), p.FOMEL02, p.FOMEL03
	*Hemitriccus margaritaceiventer*	7 (14%)	NA
	*Lathrotriccus euleri*	7 (14%)	NA
	*Sittasomus griseicapillus*	24 (42%)	NA
	*Thamnophilus pelzelni*	43 (44%)	p.THPEL01 (2), h.p.PAPOL07
	*Tolmomyias flaviventris*	21 (14%)	NA
Habitat-generalist	*Coryphospingus pileatus*	56 (62%)	h.h.COPIC01, h.p.COPIL01, h.p.PAPOL07, *P*. *nucleophilum*, p.BAFLA03 (8), p.BAFLA04 (2), p.BAHYP01, p.PADOM11
		32 (59%)	p.BAFLA03 (3), p.BAFLA04 (2), p.PHPAT01

^a^ Overall *Plasmodium/Haemoproteus* prevalence assessed by PCR following Fallon et al. (2003).

^b^ Cyt b sequencing following Hellgren et al. (2004). p. = *Plasmodium* spp.; h.h. = *Haemoproteus* (*Haemoproteus*) spp.; h.p. = *Haemoproteus* (*Parahaemoproteus*) spp. NA = No sequences obtained.

Parasite communities were homogenously distributed across successional stages as revealed by the NMDS analysis (Fig 2B), with no difference in parasite communities between successional stages (ANOSIM; R = 0.1; *P* = 0.25), suggesting that parasites may be less limited by successional stages than their hosts. Moreover, there was no correlation between the dissimilarity of bird and parasite communities across successional stages (Mantel correlation test; r = 0.18; *P* = 0.1).

**Fig 4:**
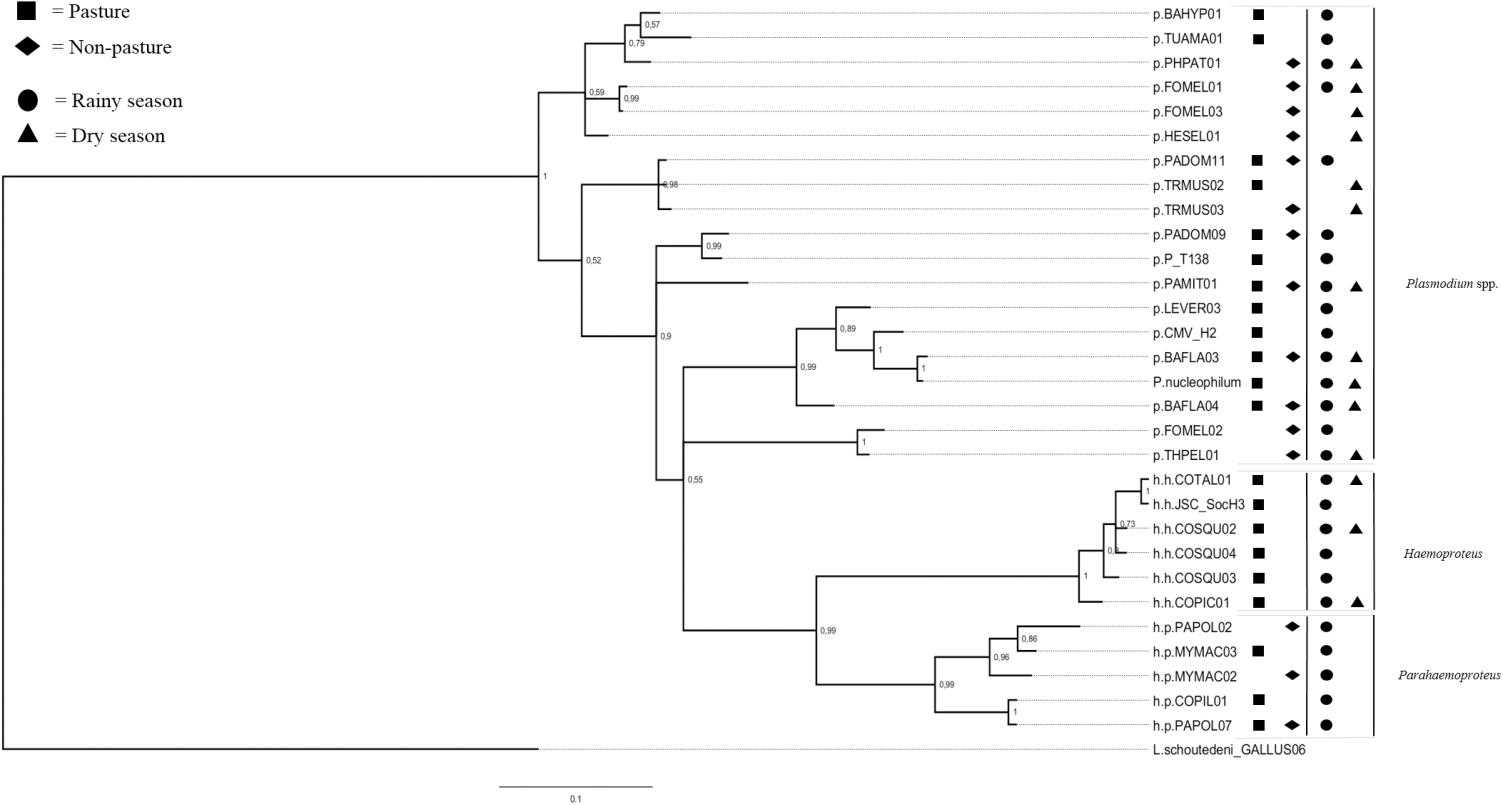
Bayesian phylogenetic tree showing lineages detected in the pasture and non-pasture areas, and in the rainy and dry seasons. Posterior probabilities and nucleotide changes (scale bar) are shown. *Leucocytozoon schoutedeni* represents the outgroup.

We detected *Haemoproteus* (*Haemoproteus*) spp. parasites only in the pasture, with six unique lineages in 11 sequenced infections (Fig 4, S3 Table). Four out of five species of doves (order Columbiformes) and one species of Passeriform bird (*Coryphospingus pileatus*) were infected by this subgenus in all four sampling periods. *Haemoproteus* (*Parahaemoproteus*) parasites were detected three times in the pasture and four times in the intermediate stage, showing no clear pattern of distribution across successional stages.

### Parasites and seasonality

Total haemosporidian prevalence did not vary between the end of the rainy season and the middle of the dry season (36.2% (43/119) vs. 29.4% (30/102); χ^2^ test, *P* = 0. 36, χ^2^ = 0.8, df = 1; Table 1), but prevalence did increase at the end of the dry season (29.4% (30/102) vs. 49.2% (62/126); χ^2^ test, *P* < 0.01, χ^2^ = 8.4, df = 1; Table 1). Prevalence then remained at the same level at the peak of the following rainy season (49.2% (62/126) vs. 51.7% (59/114); χ^2^ test, *P* = 0.8, χ^2^ = 0.07, df = 1; Table 1). Parasite prevalence also increased from the middle to the end of the dry season in *Thamnophilus pelzelni* (20% (2/10) vs. 72.7% (8/11); Fisher’s exact test, *P* = 0.03; S1 Table), but did not differ for *C*. *pileatus* (48% (13/ 27) vs. 67.7% (21/31); Fisher’s exact test, *P* = 0.18; S1 Table), two species well sampled in both dry periods. To remove the effect of the *H*. (*Haemoproteus*) spp. in the seasonal analysis, we excluded Columbiform birds, and the pattern in prevalence remained the same between the middle and the end of the dry season (25.8% (24/93) vs. 47.1% (57/121); χ^2^ test, *P* < 0.01, χ^2^ = 9.3; df = 1). The *Parahaemoproteus* subgenus was not detected during the dry season.

We also examined the effect of season on the prevalence of the most common *Plasmodium* lineage, BAFLA03 (n = 21). BAFLA03 infected eight species in four families from the order Passeriformes. Prevalence of this lineage at the end of the rainy season was 16.7% (2/12) and remained at the same level towards the middle of the dry season (31.25% (5/16); Fisher’s exact test; *P* = 0.66). The prevalence of BAFLA03 did not vary by the end of the dry season (61.1% (11/18); χ^2^ test, *P* = 0.16, χ^2^ = 1.95, df = 1; S2 Fig), but it did decrease at the peak of the rainy season (8.6% (3/35); Fisher’s Exact Test; *P* < 0.001; S2 Fig).

Overall, 10 parasite lineages were detected at the end of the rainy season; eight and seven lineages were detected at the middle and at the end of the dry season, respectively, and 21 parasite lineages were detected at the peak of the following rainy season (S3 Table).

The lineage PADOM11 was detected six times at the peak of the rainy season only, even though three out of four of its hosts (*Camptostoma obsoletum*, *C*. *pileatus*, and *V*. *jacarina*) were captured year-round. From the subgenus *Haemoproteus* (*Parahaemoproteus*), one lineage was detected twice at the end of the rainy season and five different lineages were detected at the peak of the following rainy season.

## Discussion

Deforestation events have created mosaics of secondary forests at different successional stages in tropical biomes across the globe. Therefore, it is important to understand how host-parasite interactions may change in forests undergoing secondary succession. Here we found differences in bird communities between pasture and non-pasture areas (secondary forests) in a SDTF. However, parasite communities were similar among successional stages, suggesting that parasite distributions are less restricted to habitat than the distributions of their hosts in this study area. Furthermore, we found higher haemosporidian prevalence in bird communities captured in pasture areas compared with communities from non-pasture areas.

Bird species captured exclusively in the pasture areas had higher prevalence than species captured exclusively in non-pasture areas. Indeed, animal species that act as primary pathogen reservoirs commonly are resilient to habitat modification, amplifying disease transmission in disturbed areas [20,22,61]. Our results are consistent with the idea that pasture-specialists are important reservoirs of heamosporidians and may increase local transmission. *Volatinia jacarina*, *Ammodramus humeralis* and *Columbina picui* inhabit pasture areas of SDTF at high population densities, where they build nests at the ground level, and obtain grass seeds [62]. These pasture-specialists were found to be infected by four of the most host-generalist *Plasmodium* lineages detected in Southeastern Brazil (see specificity indexes for BAFLA03, PADOM11, *P*. *cathemerium* (PADOM09), and *P*. *nucleophilum* (DENPET03) in Pinheiro et al. [63]) and were more often infected than forest-specialists. This increased abundance of infected resilient hosts and the low host specificity of the parasites they harbor may increase local transmission [64,65], explaining the overall higher prevalence of haemosporidians in pasture areas. Increased host density is associated to higher prevalence of vector-borne diseases [66], what may explain the overall higher prevalence in pasture areas regardless the influence exerted by the presence of pasture-specialists birds. Therefore, the high prevalence in pasture-specialists can simply be a result of the overall prevalence in pasture areas, and not be causative. Our sampling method did not allow us to determine bird abundances confidently in each successional stage, however, the pasture areas were the successional stage with the highest number of sampled birds.

Alternatively, or additionally, to the hypotheses mentioned above, vector abundance and distribution may also play a role in parasite prevalence in hosts at different successional stages. In a separate analysis of this area, we found a higher abundance of putative vectors of avian *Plasmodium* and higher abundance of total mosquito species in pasture areas when compared with other successional stages [67]. This indicates that higher vector abundance may pose greater risks of infection by these parasites, as has been demonstrated in human malaria [68]. We do not know the distribution of Ceratopogonidae flies in our study area, but transmission of *Parahaemoproteus* occur in forested areas (non-pasture), as forest-specialists birds were found infected by this subgenus. On the other hand, we cannot confirm that these parasites are transmitted in the pasture areas, as no pasture-specialist birds were found infected, although habitat-generalist species infected by *Parahaemoproteus* (*Coryphospingus pileatus* and *Myiodynastes maculatus*) were sampled in this successional stage.

Half of the parasite lineages detected in those three avian species restricted to pasture areas were also detected in *Coryphospingus pileatus* (the bird species well-sampled in all stages), including the generalist *Plasmodium* parasites, lineages BAFLA03 and PADOM11, and *P*. *nucleophilum*. This bird species is tolerant to disturbed habitats but was commonly found in areas at advanced successional stages as well. Prevalence in *C*. *pileatus* was considerably high in all areas (60%, 62%, 52% and 60% at pasture, early, intermediate and late stages, respectively), showing that this species may be important for parasite dispersal among successional stages, given its broad habitat distribution. This abundant species forages at the ground level in mixed flocks in pasture and forested areas [62], which may facilitate interspecific haemosporidian transmission. This may explain the homogenous distribution of parasite lineages among different successional stages despite the fact that avian communities were dissimilar between pasture and non-pasture areas.

Interestingly, bird and parasite distributions were not correlated across successional stages. Birds had a more structured distribution across successional stages even at this small geographic scale, with a clear distinction between species sampled in the pasture and non-pasture areas. Conversely, haemosporidians were homogenously distributed, suggesting that they move more freely than their hosts across successional stages. The proximity of our sample areas implies relatively small or nonexistent differences in certain abiotic factors (e.g., rainfall, temperature, and altitude) among successional stages. Consequently, such factors did not influence avian distribution in our study, but they are known to influence the prevalence of avian haemosporidians [29,69]. Ultimately, our geographically fine-scale sampling allowed us to control for these abiotic factors and evaluate the effect of the secondary successional stage itself on hosts and parasites distributions. Thus, our findings can be attributed mainly to different biotic factors among the successional stages, such as vegetation composition and structure and perhaps vector distributions. These vegetation aspects change rapidly in the beginning of the secondary succession in SDTFs when compared with other tropical ecosystems [39,40], and may explain differences in the avian community between pasture areas and all other non-pasture areas.

Similar studies have been carried out in tropical forests around the world. For example, haemosporidian prevalence was higher in more intact forests of Cameroon [28,29] and in Ghana [30] than in disturbed forests, and *Haemoproteus* prevalence was higher in continuous forests when compared with fragmented forests in Australia [31]. However, one of the two bird species assessed by Chasar et al. [29] in Cameroon were more infected by some *Plasmodium* parasites in disturbed areas. In the SDTF we investigated, the progression of secondary succession was associated with reduced haemosporidian prevalence. These contrasting results in studies across tropical forests corroborate previous studies highlighting the notion that altered ecosystems can shape host-parasite interactions idiosyncratically at local scales [69].

Since we did not detect *Haemoproteus* (*Parahaemoproteus*) spp. in the dry season, it is likely that *Plasmodium* spp. were responsible for the increase in prevalence at the end of the dry season. Our mist-netting during the end of the dry season (last week of September 2013) corresponded to one month after the beginning of the breeding season of birds in southeastern Brazil [70,71]. The increase in *Plasmodium* prevalence in birds may therefore be associated with the avian host reproductive period [72–74], which is related to “spring relapse” (i.e., an increase in parasite’s multiplication rates via merogony at tissue levels after a latent stage of infection, with a significant increase in parasitemia) in temperate latitudes [74,75]. Hydric resources, such as bodies of water scattered throughMata Seca State Park, are limited at the end of the dry season (unpublished observations). Moreover, food availability is limited during this season as well, mainly due to the reduction of fruits, seeds [40], and insects [41] that constitute important nutrient resources for these birds. Since avian nutritional status is important for controlling malaria infections [76], the marked seasonality of this ecosystem may also influence the increase in haemosporidian prevalence we observed. The seasonal effect on parasite prevalence found here is not general, since one out of the two well-sampled species did not follow this pattern (*C*. *pileatus*), depicting similar prevalence throughout the sampling periods. A few studies have addressed seasonality of avian haemosporidians in Neotropical forests, and they found no difference in overall prevalence [77,78] and in parasite lineages distributions [77] when comparing birds sampled in dry and rainy seasons. However, we did find evidence of a seasonal effect in a single well-sampled parasite lineage (BAFLA03), which accounted for 61% of the detected lineages at the end of the dry season, and 8.5% of lineages detected in the peak of the following rainy season.

We did not detect *Parahaemoproteus* during the dry season, showing that this subgenus also has marked seasonality in this study area. This subgenus can be restricted to the internal organs of its hosts at certain periods of the year, and may therefore be difficult to detect in the blood stream [79,80]. The seasonal restriction for *Plasmodium* and *Parahaemoproteus* does not follow the same pattern: the former was detected year round, only changing overall prevalence within the dry season, while the latter was not detected during the dry season.

We detected *Haemoproteus* (*Haemoproteus*) spp. only in pasture areas, and this was likely due to the markedly higher presence of Columbiform birds in this successional stage. A passerine bird (*C*. *pileatus*) captured in the pasture was also infected with *H*. (*Haemoproteus*) spp., and this parasite subgenus has also been found in a passerine in another part of southeastern Brazil [81], suggesting that Hippoboscid flies also feed upon non-Columbiforms in Brazil. These likely were abortive infections [82], given that this subgenus completes its life cycle only in Columbiforms and in some marine birds [83]. Interestingly, the *H*. (*Haemoproteus*) subgenus was detected during the dry season, showing that their vectors may persist year-round. In fact, Hippoboscid flies spend almost all of their adult life on their host [84], making parasite transmission possible year-round at mid to high latitudes [74].

In conclusion, we have shown that secondary succession in SDTFs is associated with compositional differences in avian communities, and that advanced successional stages are associated with lower prevalence of avian haemosporidian parasites. We also found effects of seasonality on parasite prevalence and in the relative abundances of specific lineages. These seasonal effects may be related to the hosts’ breeding phenology, which overlaps with a marked dry season that limits food and water resources. Further studies in seasonally dry tropical forests may reveal if the seasonal pattern of infection that we found is general or restricted to a single season in our area of study. Nevertheless, our results warrant further periodic investigations in tropical forests to understand temporal drivers of parasite ecology in these environments. Finally, understanding the effects of forest recovery on the dispersal of parasites may help to formulate conservation strategies to improve environmental and animal health.

## Supporting information

S1 Fig Haemosporidian prevalence in specialists birds when captured in pasture and in non-pasture areas, respectively. Pasture-specialists: *Ammodramus humeralis* (n = 8), *Columbina picui* (n = 8), *Volatinia jacarina* (n = 27). Forest-specialists: *Basileuterus flaveolus* (n = 8), *Cnemotriccus fuscatus* (n = 10), *Formicivora melanogaster* (n = 19), *Hemitriccus margaritaceiventer* (n = 7), *Lathrotriccus euleri* (n = 7), *Sittasomus griseicapillus* (n = 24), *Thamnophilus pelzelni* (n = 43), *Tolmomyias flaviventris* (n = 21). χ^2^ test, *P* = 0.01, χ^2^ = 6.2; df = 1.
(TIF)

S2 Fig Seasonal variation of the relative prevalence of the Plasmodium lineage BAFLA03. Numbers in parenthesis represent the total of BAFLA03 sequences obtained in each period relative to the number of overall parasite (*Plasmodium and Haemoproteus*) lineages.
(TIF)

S1 Table Number of individuals captured and haemosporidian prevalence by season. **Only species sampled four or more times are included.** Numbers represent sample size. a = well-sampled species captured in all season; b = well-sampled species captured only in the rainy season.
(DOCX)

S2 Table Description of captured birds and haemosporidian prevalence per successional stage. **Only species sampled four or more times were included.** Numbers represent sample size. a = well-sampled species captured only in early, intermediate and late stages; b well-sampled species captured only in pasture areas; c = well-sampled species with three or more individuals captured in the pasture and three or more individuals captured in the remaining stages; d = well-sampled species that do not meet any of those criteria.
(DOCX)

S3 Table Haemosporidian lineages detected according to successional stage and season of sampling. n = Number of times that the association between lineage/species/season was found. h.h. = *Haemoproteus* (*Haemoproteus*) spp.; h.p. = *Haemoproteus* (*Parahaemoproteus*) spp., p. = *Plasmodium*. Lineage names in bold represent lineages with no previous description.
(DOCX)
