# Supplementary figures and images for: Habitat modification and seasonality influence avian haemosporidian parasite distributions in southeastern Brazil
Source: PLoS One. 2017 Jun 2;12(6):e0178791. doi: 10.1371/journal.pone.0178791 (PMC5456369; doi:10.1371/journal.pone.0178791)

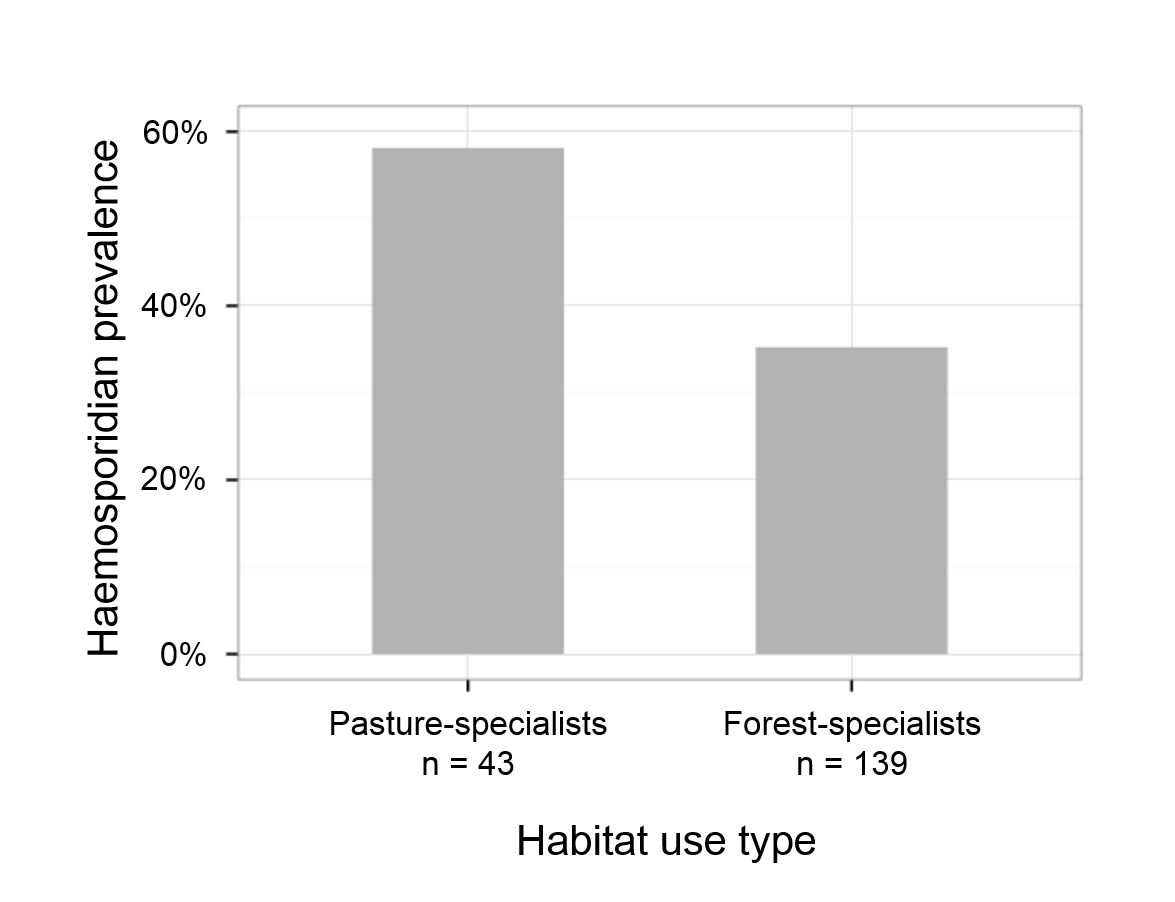

Supplement: S1 Fig — Pasture-specialists: Ammodramus humeralis (n = 8), Columbina picui (n = 8), Volatinia jacarina (n = 27). Forest-specialists: Basileuterus flaveolus (n = 8), Cnemotriccus fuscatus (n = 10), Formicivora melanogaster (n = 19), Hemitriccus margaritaceiventer (n = 7), Lathrotriccus euleri (n = 7), Sittasomus griseicapillus (n = 24), Thamnophilus pelzelni (n = 43), Tolmomyias flaviventris (n = 21). χ2 test, P = 0.01, χ2 = 6.2; df = 1. (TIF) [file pone.0178791.s001.tif]

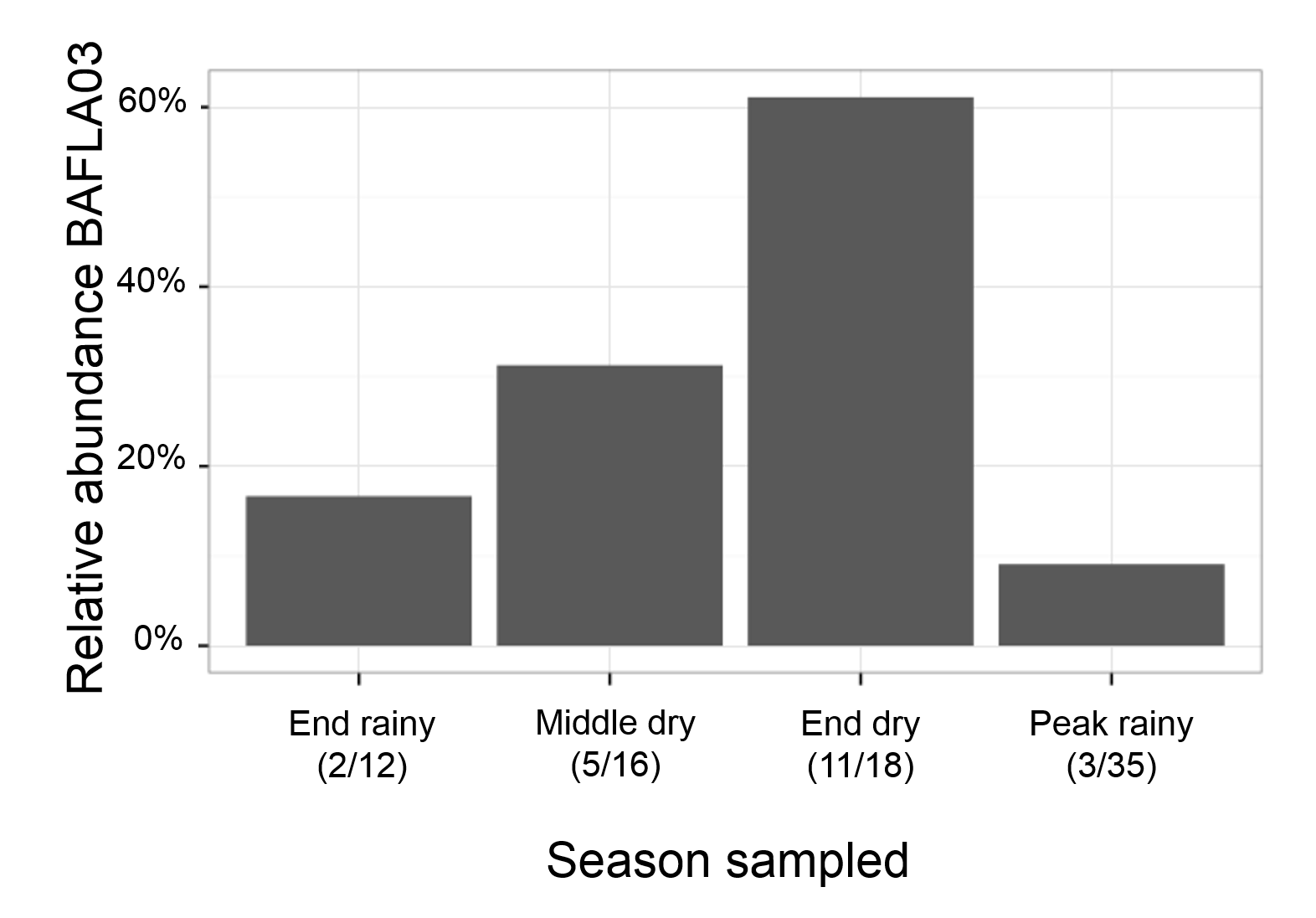

Supplement: S2 Fig — Numbers in parenthesis represent the total of BAFLA03 sequences obtained in each period relative to the number of overall parasite (Plasmodium and Haemoproteus) lineages. (TIF) [file pone.0178791.s002.tif]
