# Supplementary material for: Habitat modification and seasonality influence avian haemosporidian parasite distributions in southeastern Brazil
Source: PLoS One. 2017 Jun 2;12(6):e0178791. doi: 10.1371/journal.pone.0178791 (PMC5456369; doi:10.1371/journal.pone.0178791)
Supplement: S1 Table — Only species sampled four or more times are included. Numbers represent sample size. a = well-sampled species captured in all season; b = well-sampled species captured only in the rainy season. (DOCX) [file pone.0178791.s003.docx]

**S1 Table: Number of individuals captured and haemosporidian prevalence by season.**

| **Species** | **Family** | **End rainy (%)** | | **Middle dry (%)** | | **End dry (%)** | | **Peak rainy (%)** | | **Total (%)** | |
| --- | --- | --- | --- | --- | --- | --- | --- | --- | --- | --- | --- |
| *Nystalus maculatus* | Buconidae (Galbuliformes) | 1 | 0% | 5 | 20% | 4 | 25% | 4 | 0% | 14 | 14% |
| *Columbina minuta* | Columbidae (Columbiformes) | 1 | 0% | 0 | NA | 0 | NA | 1 | 100% | 2 | 50% |
| *Columbina picui* | Columbidae (Columbiformes) | 1 | 0% | 4 | 75% | 1 | 100% | 2 | 50% | 8 | 63% |
| *Columbina squammata* | Columbidae (Columbiformes) | 1 | 0% | 2 | 50% | 3 | 100% | 6 | 83% | 12 | 75% |
| *Columbina talpacoti* | Columbidae (Columbiformes) | 0 | NA | 2 | 100% | 0 | NA | 1 | 100% | 3 | 100% |
| *Leptotila verreauxi* | Columbidae (Columbiformes) | 2 | 50% | 1 | 0% | 1 | 100% | 0 | NA | 4 | 50% |
| *Cyanocorax cyanopogon* | Corvidae | 1 | 0% | 0 | NA | 2 | 50% | 0 | NA | 3 | 33% |
| *Campylorhamphus trochilirostris* | Dendrocolaptidae | 0 | NA | 2 | 0% | 0 | NA | 1 | 100% | 1 | 100% |
| *Dendrocolaptes platyrostris* | Dendrocolaptidae | 2 | 0% | 0 | NA | 2 | 0% | 0 | NA | 4 | 0% |
| *Lepidocolaptes angustirostris* | Dendrocolaptidae | 2 | 0% | 0 | NA | 1 | 0% | 1 | 0% | 4 | 0% |
| *Lepidocolaptes wagleri* | Dendrocolaptidae | 0 | NA | 1 | 0% | 1 | 0% | 0 | NA | 2 | 0% |
| *Sittasomus griseicapillus* | Dendrocolaptidae | 12 | 50% | 4 | 0% | 2 | 50% | 6 | 50% | 24 | 42% |
| *Furnarius leucopus* | Furnariidae | 0 | NA | 1 | 0% | 3 | 66% | 0 | NA | 4 | 50% |
| *Synallaxis albescens* | Furnariidae | 0 | NA | 0 | NA | 0 | NA | 1 | 100% | 1 | 100% |
| *Synallaxis frontalis* | Furnariidae | 0 | NA | 0 | NA | 0 | NA | 1 | 0% | 1 | 0% |
| *Synallaxis scutata* | Furnariidae | 0 | NA | 2 | 50% | 0 | NA | 3 | 33% | 5 | 40% |
| *Myiobius barbatus* | Onychorhynchidae | 0 | NA | 1 | 0% | 1 | 0% | 0 | NA | 2 | 0% |
| *Basileuterus flaveolus* | Parulidae | 2 | 0% | 1 | 0% | 2 | 50% | 3 | 66% | 8 | 38% |

**Cont. S1 Table.**

| **Species** | **Family** | **End rainy (%)** | | **Middle dry (%)** | | **End dry (%)** | | **Peak rainy (%)** | | **Total (%)** | |
| --- | --- | --- | --- | --- | --- | --- | --- | --- | --- | --- | --- |
| *Ammodramus humeralis* | Passerelidae | 1 | 0% | 0 | NA | 1 | 100% | 6 | 66% | 8 | 63% |
| *Colaptes melanochloros* | Picidae | 0 | NA | 0 | NA | 0 | NA | 1 | 100% | 1 | 100% |
| *Picumnus pygmaeus* | Picidae | 0 | NA | 0 | NA | 1 | 0% | 0 | NA | 1 | 0% |
| *Polioptila plumbea* | Polioptilidae | 1 | 100% | 1 | 0% | 1 | 0% | 0 | NA | 3 | 33% |
| *Aratinga cactorum* | Psittacidae | 0 | NA | 0 | NA | 0 | NA | 2 | 50% | 2 | 50% |
| *Hemitriccus margaritaceiventer* | Rhynchocyclidae | 3 | 0% | 4 | 25% | 0 | NA | 0 | NA | 7 | 14% |
| *Todirostrum cinereum* | Rhynchocyclidae | 0 | NA | 1 | 0% | 0 | NA | 0 | NA | 1 | 0% |
| *Tolmomyias flaviventris* | Rhynchocyclidae | 5 | 40% | 6 | 100% | 7 | 0% | 3 | 33% | 21 | 14% |
| *Tolmomyias sulphurescens* | Rhynchocyclidae | 0 | NA | 1 | 0% | 3 | 0% | 1 | 0% | 5 | 0% |
| *Formicivora melanogaster* | Thamnophilidae | 4 | 25% | 8 | 25% | 3 | 100% | 4 | 50% | 19 | 42% |
| *Herpsilochmus sellowi* | Thamnophilidae | 0 | NA | 0 | NA | 2 | 50% | 0 | NA | 2 | 50% |
| *Sakesphorus cristatus* | Thamnophilidae | 0 | NA | 1 | 100% | 4 | 75% | 1 | 100% | 6 | 83% |
| *Thamnophilus capistratus* | Thamnophilidae | 0 | NA | 3 | 0% | 2 | 100% | 0 | NA | 5 | 40% |
| *Thamnophilus doliatus* | Thamnophilidae | 0 | NA | 0 | NA | 1 | 100% | 0 | NA | 1 | 100% |
| *Thamnophilus pelzelni^a^* | Thamnophilidae | 13 | 46% | 10 | 20% | 11 | 72% | 9 | 33% | 43 | 44% |
| *Coereba flaveola* | Thraupidae | 0 | NA | 1 | 100% | 0 | NA | 0 | NA | 1 | 100% |
| *Conirostrum speciosum* | Thraupidae | 3 | 0% | 2 | 0% | 1 | 0% | 1 | 0% | 7 | 0% |
| *Coryphospingus pileatus^a^* | Thraupidae | 13 | 53% | 27 | 48% | 31 | 67% | 19 | 63% | 90 | 59% |
| *Paroaria dominicana* | Thraupidae | 1 | 100% | 0 | NA | 2 | 50% | 1 | 0% | 4 | 50% |
| *Sporophila nigricollis* | Thraupidae | 1 | 100% |  | 0 | NA | 0 | NA | 0 | 1 | 100% |

**Cont. S1 Table.**

| **Species** | **Family** | **End rainy (%)** | | **Middle dry (%)** | | **End dry (%)** | | **Peak rainy (%)** | | **Total (%)** | |
| --- | --- | --- | --- | --- | --- | --- | --- | --- | --- | --- | --- |
| *Thlypopsis sordida* | Thraupidae | 1 | 100% |  | 0 | NA | 0 | NA | 0 | 1 | 100% |
| *Volatinia jacarina* | Thraupidae | 4 | 75% | 1 | 0% | 8 | 37% | 15 | 60% | 28 | 54% |
| *Pachyramphus polychopterus* | Tityridae | 2 | 50% | 0 | NA | 0 | NA | 1 | 100% | 3 | 66% |
| *Troglodytes musculus* | Troglodytidae | 1 | 0% | 4 | 50% | 2 | 0% | 0 | NA | 7 | 29% |
| *Turdus albicollis* | Turdidae | 1 | 0% | 0 | NA | 1 | 0% | 0 | NA | 2 | 0% |
| *Turdus amaurochalinus* | Turdidae | 5 | 20% | 0 | NA | 1 | 0% | 3 | 0% | 9 | 11% |
| *Turdus leucomelas* | Turdidae | 0 | NA | 0 | NA | 1 | 100% | 0 | NA | 1 | 100% |
| *Camptostoma obsoletum* | Tyrannidae | 1 | 0% | 1 | 0% | 1 | 0% | 2 | 50% | 5 | 20% |
| *Casiornis fuscus* | Tyrannidae | 1 | 100% | 0 | NA | 0 | NA | 0 | NA | 1 | 100% |
| *Cnemotriccus fuscatus* | Tyrannidae | 5 | 60% | 3 | 0% | 1 | 0% | 1 | 100% | 10 | 40% |
| *Euscarthmus meloryphus* | Tyrannidae | 2 | 0% | 0 | NA | 1 | 0% | 0 | NA | 3 | 0% |
| *Knipolegus franciscanus* | Tyrannidae | 0 | NA | 0 | NA | 1 | 100% | 0 | NA | 1 | 100% |
| *Lathrotriccus euleri* | Tyrannidae | 4 | 25% | 1 | 0% | 1 | 0% | 1 | 0% | 7 | 14% |
| *Myiarchus tyrannulus* | Tyrannidae | 1 | 0% | 1 | 0% | 9 | 44% | 0 | NA | 11 | 36% |
| *Myiarchus swainsoni* | Tyrannidae | 1 | 0% | 0 | NA | 0 | NA | 0 | NA | 1 | 0% |
| *Myiopagis viridicata^b^* | Tyrannidae | 14 | 7% | 0 | NA | 0 | NA | 5 | 40% | 19 | 16% |
| *Myiodynastes maculatus* | Tyrannidae | 2 | 50% | 0 | NA | 1 | 0% | 7 | 57% | 10 | 50% |
| *Myiophobus fasciatus* | Tyrannidae | 1 | 0% | 1 | 0% | 0 | NA | 1 | 0% | 3 | 0% |
| *Myiozetetes cayanensis* | Tyrannidae | 0 | NA | 0 | NA | 1 | 0% | 0 | NA | 1 | 0% |
| *Phaeomyias murina* | Tyrannidae | 0 | NA | 0 | NA | 0 | NA | 1 | 0% | 1 | 0% |
| *Tyrannus melancholicus* | Tyrannidae | 1 | 100% | 0 | NA | 1 | 0% | 0 | NA | 2 | 50% |

Numbers represent sample size. a = well-sampled species captured in all season; b = well-sampled species captured only in the rainy season.
