# Supplementary material for: Habitat modification and seasonality influence avian haemosporidian parasite distributions in southeastern Brazil
Source: PLoS One. 2017 Jun 2;12(6):e0178791. doi: 10.1371/journal.pone.0178791 (PMC5456369; doi:10.1371/journal.pone.0178791)
Supplement: S2 Table — Only species sampled four or more times were included. Numbers represent sample size. a = well-sampled species captured only in early, intermediate and late stages; b well-sampled species captured only in pasture areas; c = well-sampled species with three or more individuals captured in the pasture and three or more individuals captured in the remaining stages; d = well-sampled species that do not meet any of those criteria. (DOCX) [file pone.0178791.s004.docx]

**S2 Table: Description of captured birds and haemosporidian prevalence per successional stage.**

| **Species** | **Family** | **Pasture (%)** | | **Early (%)** | | **Intermediate (%)** | | **Late (%)** | | **Total (%)** | |
| --- | --- | --- | --- | --- | --- | --- | --- | --- | --- | --- | --- |
| *Nystalus maculatus^c^* | Buconidae (Galbuliformes) | 9 | 22% | 2 | 0% | 3 | 0% | 0 | NA | 14 | 14% |
| *Columbina minuta* | Columbidae (Columbiformes) | 2 | 50% | 0 | NA | 0 | NA | 0 | NA | 2 | 50% |
| *Columbina picui^b^* | Columbidae (Columbiformes) | 8 | 62% | 0 | NA | 0 | NA | 0 | NA | 8 | 63% |
| *Columbina squammata^c^* | Columbidae (Columbiformes) | 9 | 77% | 0 | NA | 2 | 50% | 1 | 100% | 12 | 75% |
| *Columbina talpacoti* | Columbidae (Columbiformes) | 3 | 100% | 0 | NA | 0 | NA | 0 | NA | 3 | 100% |
| *Leptotila verreauxi* | Columbidae (Columbiformes) | 2 | 50% | 1 | 0% | 0 | NA | 1 | 100% | 4 | 50% |
| *Cyanocorax cyanopogon* | Corvidae | 0 | NA | 3 | 33% | 0 | NA | 0 | NA | 3 | 33% |
| *Campylorhamphus trochilirostris* | Dendrocolaptidae | 0 | NA | 0 | NA | 1 | 100% | 0 | NA | 1 | 100% |
| *Dendrocolaptes platyrostris* | Dendrocolaptidae | 0 | NA | 1 | 0% | 2 | 0% | 1 | 0% | 4 | 0% |
| *Lepidocolaptes angustirostris* | Dendrocolaptidae | 1 | 0% | 3 | 0% | 0 | NA | 0 | NA | 4 | 0% |
| *Lepidocolaptes wagleri* | Dendrocolaptidae | 0 | NA | 0 | NA | 1 | 0% | 1 | 0% | 2 | 0% |
| *Sittasomus griseicapillus^a^* | Dendrocolaptidae | 0 | NA | 4 | 50% | 13 | 46% | 7 | 28% | 24 | 42% |
| *Furnarius leucopus* | Furnariidae | 0 | NA | 3 | 66% | 1 | 0% | 0 | NA | 4 | 50% |
| *Synallaxis albescens* | Furnariidae | 1 | 100% | 0 | NA | 0 | NA | 0 | NA | 1 | 100% |
| *Synallaxis frontalis* | Furnariidae | 1 | 0% | 0 | NA | 0 | NA | 0 | NA | 1 | 0% |
| *Synallaxis scutata* | Furnariidae | 0 | NA | 0 | NA | 2 | 50% | 3 | 33% | 5 | 40% |
| *Myiobius barbatus* | Onychorhynchidae | 0 | NA | 1 | 0% | 1 | 0% | 0 | NA | 2 | 0% |
| *Basileuterus flaveolus^a^* | Parulidae | 0 | NA | 1 | 0% | 3 | 33% | 4 | 50% | 8 | 38% |
| *Ammodramus humeralis^b^* | Passerelidae | 8 | 62% | 0 | NA | 0 | NA | 0 | NA | 8 | 63% |
| *Colaptes melanochloros* | Picidae | 1 | 100% | 0 | NA | 0 | NA | 0 | NA | 1 | 100% |
| *Picumnus pygmaeus* | Picidae | 0 | NA | 0 | NA | 0 | NA | 1 | 0% | 1 | 0% |

**Cont. S2 Table**

| **Species** | **Family** | **Pasture (%)** | | | **Early (%)** | | **Intermediate (%)** | | **Late (%)** | | **Total (%)** | |
| --- | --- | --- | --- | --- | --- | --- | --- | --- | --- | --- | --- | --- |
| *Polioptila plumbea* | Polioptilidae | 1 | 100% | | 0 | NA | 2 | 0% | 0 | NA | 3 | 33% |
| *Aratinga cactorum* | Psittacidae | 1 | 100% | | 1 | 0% | 0 | NA | 0 | NA | 2 | 50% |
| *Hemitriccus^a^ margaritaceiventer* | Rhynchocyclidae | 0 | NA | | 3 | 0% | 3 | 33% | 1 | 0% | 7 | 14% |
| *Todirostrum cinereum* | Rhynchocyclidae | 1 | 0% | | 0 | NA | 0 | NA | 0 | NA | 1 | 0% |
| *Tolmomyias flaviventris^a^* | Rhynchocyclidae | 0 | NA | | 1 | 0% | 10 | 10% | 10 | 20% | 21 | 14% |
| *Tolmomyias sulphurescens* | Rhynchocyclidae | 0 | NA | | 0 | NA | 1 | 0% | 4 | 0% | 5 | 0% |
| *Formicivora melanogaster^a^* | Thamnophilidae | 0 | NA | | 4 | 50% | 7 | 42% | 8 | 37% | 19 | 42% |
| *Herpsilochmus sellowi* | Thamnophilidae | 1 | 0% | | 0 | NA | 1 | 100% | 0 | NA | 2 | 50% |
| *Thamnophilus capistratus* | Thamnophilidae | 0 | NA | | 1 | 100% | 3 | 33% | 1 | 0% | 5 | 40% |
| *Thamnophilus doliatus* | Thamnophilidae | 0 | NA | | 0 | NA | 1 | 100% | 0 | NA | 1 | 100% |
| *Thamnophilus pelzelni^a^* | Thamnophilidae | 0 | NA | | 9 | 66% | 18 | 27% | 16 | 50% | 43 | 44% |
| *Sakesphorus cristatus* | Thamnophilidae | 0 | NA | | 2 | 100% | 2 | 50% | 2 | 100% | 6 | 83% |
| *Coereba flaveola* | Thraupidae | 0 | | NA | 0 | NA | 1 | 100% | 0 | NA | 1 | 100% |
| *Conirostrum speciosum^c^* | Thraupidae | 1 | 0% | | 2 | 0% | 1 | 0% | 3 | 0% | 7 | 0% |
| *Coryphospingus pileatus^c^* | Thraupidae | 56 | 60% | | 8 | 62% | 21 | 52% | 5 | 60% | 90 | 59% |
| *Paroaria dominicana* | Thraupidae | 2 | 0% | | 2 | 100% | 0 | NA | 0 | NA | 4 | 50% |
| *Sporophila nigricollis* | Thraupidae | 1 | 100% | | NA | 0 | NA | 0 | NA | 0 | 1 | 100% |
| *Thlypopsis sordida* | Thraupidae | 1 | 100% | | NA | 0 | NA | 0 | NA | 0 | 1 | 100% |
| *Volatinia jacarina^b^* | Thraupidae | 27 | 55% | | 1 | 0% | 0 | NA | 0 | NA | 28 | 54% |

**Cont. S2 Table**

| **Species** | **Family** | **Pasture (%)** | | **Early (%)** | | **Intermediate (%)** | | **Late (%)** | | **Total (%)** | |
| --- | --- | --- | --- | --- | --- | --- | --- | --- | --- | --- | --- |
| *Pachyramphus polychopterus* | Tityridae | 0 | NA | 1 | 0% | 2 | 100 | 0 | NA | 3 | 66% |
| *Troglodytes musculus^c^* | Troglodytidae | 2 | 50% | 0 | NA | 2 | 50% | 3 | 0% | 7 | 29% |
| *Turdus albicollis* | Turdidae | 0 | NA | 0 | NA | 0 | NA | 2 | 0% | 2 | 0% |
| *Turdus amaurochalinus^c^* | Turdidae | 3 | 33% | 3 | 0% | 1 | 0% | 2 | 0% | 9 | 11% |
| *Turdus leucomelas* | Turdidae | 0 | NA | 1 | 100% | 0 | NA | 0 | NA | 1 | 100% |
| *Camptostoma obsoletum* | Tyrannidae | 3 | 33% | 1 | 0% | 0 | NA | 1 | 0% | 5 | 20% |
| *Casiornis fuscus* | Tyrannidae | 0 | NA | 1 | 100% | 0 | NA | 0 | NA | 1 | 100% |
| *Cnemotriccus fuscatus^a^* | Tyrannidae | 0 | NA | 2 | 0% | 4 | 50% | 4 | 50% | 10 | 40% |
| *Euscarthmus meloryphus* | Tyrannidae | 3 | 0% | 0 | NA | 0 | NA | 0 | NA | 3 | 0% |
| *Knipolegus franciscanus* | Tyrannidae | 0 | NA | 1 | 0% | 0 | NA | 0 | NA | 1 | 0% |
| *Lathrotriccus euleri^a^* | Tyrannidae | 0 | NA | 3 | 0% | 1 | 0% | 3 | 33% | 7 | 14% |
| *Myiopagis viridicata^c^* | Tyrannidae | 1 | 0% | 5 | 40% | 4 | 0% | 9 | 11% | 19 | 16% |
| *Myiarchus tyrannulus^c^* | Tyrannidae | 5 | 60% | 3 | 33% | 1 | 0% | 2 | 0% | 11 | 36% |
| *Myiarchus swainsoni* | Tyrannidae | 0 | NA | 1 | 0% | 0 | NA | 0 | NA | 1 | 0% |
| *Myiodynastes maculatus^c^* | Tyrannidae | 6 | 66% | 0 | NA | 1 | 100% | 3 | 0% | 10 | 50% |
| *Myiophobus fasciatus* | Tyrannidae | 3 | 0% | 0 | NA | 0 | NA | 0 | NA | 3 | 0% |
| *Myiozetetes cayanensis* | Tyrannidae | 1 | 100% | 0 | NA | 0 | NA | 0 | NA | 1 | 100% |
| *Phaeomyias murina* | Tyrannidae | 0 | NA | 0 | NA | 0 | NA | 1 | 0% | 1 | 0% |
| *Tyrannus melancholicus* | Tyrannidae | 2 | 50% | 0 | NA | 0 | NA | 0 | NA | 2 | 50% |

Numbers represent sample size. a = well-sampled species captured only in early, intermediate and late stages; b well-sampled species captured only in pasture areas; c = well-sampled species that do not meet any of those criteria.
