# Supplementary material for: Habitat modification and seasonality influence avian haemosporidian parasite distributions in southeastern Brazil
Source: PLoS One. 2017 Jun 2;12(6):e0178791. doi: 10.1371/journal.pone.0178791 (PMC5456369; doi:10.1371/journal.pone.0178791)
Supplement: S3 Table — n = Number of times that the association between lineage/species/season was found. h.h. = Haemoproteus (Haemoproteus) spp.; h.p. = Haemoproteus (Parahaemoproteus) spp., p. = Plasmodium. Lineage names in bold represent lineages with no previous description. (DOCX) [file pone.0178791.s005.docx]

**S3 Table: Haemosporidian lineages detected according to successional stage and season of sampling.**

| **Lineage** | **Species** | **Successional stage** | **Season** | **n** | **GenBank acc. No.** |
| --- | --- | --- | --- | --- | --- |
| **h.h.COSQU02** | *Columbina squammata* | Pasture | End dry | 1 | KP686107 |
| **h.h.COSQU02** | *Columbina squammata* | Pasture | Peak rainy | 2 | KP686107 |
| **h.h.COSQU03** | *Columbina squammata* | Pasture | Peak rainy | 1 | KP686098 |
| **h.h.COSQU04** | *Columbina squammata* | Pasture | Peak rainy | 1 | KP686099 |
| h.h.COTAL01 | *Columbina talpacoti* | Pasture | Peak rainy | 1 | KY304994 |
| h.h.COTAL01 | *Columbina talpacoti* | Pasture | Middle dry | 1 | KY304994 |
| h.h.JSC-2012 isolate SocH3 | *Columbina minuta* | Pasture | Peak rainy | 1 | KY304995 |
| **h.h.COPIC01** | *Columbina picui* | Pasture | Peak rainy | 1 | KP686106 |
| **h.h.COPIC01** | *Columbina picui* | Pasture | Middle dry | 1 | KP686106 |
| **h.h.COPIC01** | *Coryphospingus pileatus* | Pasture | Middle dry | 1 | KP686106 |
| **h.p.COPIL01** | *Coryphospingus pileatus* | Pasture | Peak rainy | 1 | KP686096 |
| **h.p.MYMAC02** | *Myiodynastes maculatus* | Intermediate | Peak rainy | 1 | KP686097 |
| **h.p.MYMAC03** | *Myiodynastes maculatus* | Pasture | Peak rainy | 1 | KY305008 |
| h.p.PAPOL02 | *Cnemotriccus fuscatus* | Intermediate | Peak rainy | 1 | KY304996 |
| **h.p.PAPOL07** | *Coryphospingus pileatus* | Pasture | Peak rainy | 1 | KP686101 |
| **h.p.PAPOL07** | *Pachyramphus polychopterus* | Intermediate | End rainy | 1 | KP686101 |
| **h.p.PAPOL07** | *Thamnophilus pelzelni* | Intermediate | End rainy | 1 | KP686101 |
| *P. nucleophilum* | *Ammodramus humeralis* | Pasture | Peak rainy | 1 | KY346520 |
| *P. nucleophilum* | *Colaptes melanochloros* | Pasture | Peak rainy | 1 | KY346520 |
| *P. nucleophilum* | *Coryphospingus pileatus* | Pasture | Middle dry | 1 | KY346520 |
| *P. nucleophilum* | *Volatinia jacarina* | Pasture | End rainy | 1 | KY346520 |
| p.BAFLA03 | *Basileuterus flaveolus* | Late | End dry | 1 | KY304997 |
| p.BAFLA03 | *Cnemotriccus fuscatus* | Late | End rainy | 1 | KY304997 |
| p.BAFLA03 | *Coryphospingus pileatus* | Intermediate | Peak rainy | 1 | KY304997 |
| p.BAFLA03 | *Coryphospingus pileatus* | Intermediate | Middle dry | 1 | KY304997 |
| p.BAFLA03 | *Coryphospingus pileatus* | Intermediate | End dry | 1 | KY304997 |
| p.BAFLA03 | *Coryphospingus pileatus* | Pasture | Middle dry | 3 | KY304997 |
| p.BAFLA03 | *Coryphospingus pileatus* | Pasture | End dry | 5 | KY304997 |
| p.BAFLA03 | *Myiarchus tyrannulus* | Pasture | End dry | 2 | KY304997 |
| p.BAFLA03 | *Myiopagis viridicata* | Early | Peak rainy | 1 | KY304997 |
| p.BAFLA03 | *Sakesphorus cristatus* | Intermediate | Middle dry | 1 | KY304997 |
| p.BAFLA03 | *Thlypopsis sordida* | Pasture | End rainy | 1 | KY304997 |
| p.BAFLA03 | *Volatinia jacarina* | Pasture | End dry | 2 | KY304997 |
| p.BAFLA03 | *Volatinia jacarina* | Pasture | Peak rainy | 1 | KY304997 |
| p.BAFLA04 | *Coereba flaveola* | Intermediate | Middle dry | 1 | KY304998 |
| p.BAFLA04 | *Columbina squammata* | Pasture | Peak rainy | 1 | KY304998 |
| p.BAFLA04 | *Coryphospingus pileatus* | Pasture | Peak rainy | 1 | KY304998 |
| p.BAFLA04 | *Coryphospingus pileatus* | Intermediate | Middle dry | 1 | KY304998 |
| p.BAFLA04 | *Coryphospingus pileatus* | Late | Middle dry | 1 | KY304998 |
| p.BAFLA04 | *Coryphospingus pileatus* | Pasture | Middle dry | 1 | KY304998 |
| p.BAHYP01 | *Coryphospingus pileatus* | Pasture | End rainy | 1 | KY304999 |

**Cont. S3 Table**

| **Lineage** | **Species** | **Successional stage** | **Season** | **n** | **GenBank acc. No.** |
| --- | --- | --- | --- | --- | --- |
| p.CMV-2012 haplotype H2 | *Myiodynastes maculatus* | Pasture | Peak rainy | 1 | KY305000 |
| p.FOMEL01 | *Formicivora melanogaster* | Late | Peak rainy | 1 | KY305001 |
| p.FOMEL01 | *Formicivora melanogaster* | Intermediate | Middle dry | 1 | KY305001 |
| **p.FOMEL02** | *Formicivora melanogaster* | Late | End rainy | 1 | KP686102 |
| **p.FOMEL03** | *Formicivora melanogaster* | Early | End dry | 1 | KP686108 |
| **p.HESEL01** | *Herpsilochmus sellowi* | Intermediate | End dry | 1 | KP686095 |
| **p.LEVER03** | *Leptotila verreauxi* | Pasture | End rainy | 1 | KP686103 |
| *P. cathemerium* | *Ammodramus humeralis* | Pasture | Peak rainy | 1 | KY305002 |
| *P. cathemerium* | *Cnemotriccus fuscatus* | Intermediate | End rainy | 1 | KY305002 |
| *P. cathemerium* | *Myiopagis viridicata* | Early | Peak rainy | 1 | KY305002 |
| *P. cathemerium* | *Synallaxis scutata* | Intermediate | Peak rainy | 1 | KY305002 |
| p.PADOM11 | *Camptostoma obsoletum* | Pasture | Peak rainy | 1 | KY305003 |
| p.PADOM11 | *Campylorhamphus trochilirostris* | Intermediate | Peak rainy | 1 | KY305003 |
| p.PADOM11 | *Coryphospingus pileatus* | Pasture | Peak rainy | 1 | KY305003 |
| p.PADOM11 | *Volatinia jacarina* | Pasture | Peak rainy | 3 | KY305003 |
| p.PAMIT01 | *Leptotila verreauxi* | Late | End dry | 1 | KY305004 |
| p.PAMIT01 | *Myiodynastes maculatus* | Pasture | Peak rainy | 1 | KY305004 |
| p.PHPAT01 | *Basileuterus flaveolus* | Late | Peak rainy | 1 | KY305005 |
| p.PHPAT01 | *Coryphospingus pileatus* | Intermediate | End dry | 1 | KY305005 |
| p.PHPAT01 | *Paroaria dominicana* | Early | End dry | 1 | KY305005 |
| p.P-T138 | *Volatinia jacarina* | Pasture | End rainy | 1 | KY305006 |
| p.P-T138 | *Volatinia jacarina* | Pasture | Peak rainy | 3 | KY305006 |
| **p.THPEL01** | *Thamnophilus pelzelni* | Early | End rainy | 1 | KP686094 |
| **p.THPEL01** | *Thamnophilus pelzelni* | Intermediate | End dry | 1 | KP686094 |
| **p.TRMUS02** | *Troglodytes musculus* | Pasture | Middle dry | 1 | KP686104 |
| **p.TRMUS03** | *Troglodytes musculus* | Intermediate | Middle dry | 1 | KP686105 |
| p.TUAMA01 | *Turdus amaurochalinus* | Pasture | End rainy | 1 | KY305007 |

n = Number of times that the association between lineage/species/season was found. h.h. = *H*. (*Haemoproteus*) spp.; h.p. = *H*. (*Parahaemoproteus*) spp., p. = *Plasmodium.* Lineage names in bold represent lineages with no previous description.
